# Quantitative image quality metrics enable resource-efficient quality control of clinically applied AI-based reconstructions in MRI

**DOI:** 10.1007/s10334-025-01253-3

**Published:** 2025-05-24

**Authors:** Owen A. White, Joshua Shur, Francesca Castagnoli, Geoff Charles-Edwards, Brandon Whitcher, David J. Collins, Matthew T. D. Cashmore, Matt G. Hall, Spencer A. Thomas, Andrew Thompson, Ciara A. Harrison, Georgina Hopkinson, Dow-Mu Koh, Jessica M. Winfield

**Affiliations:** 1 MRI Unit, The Royal Marsden NHS Foundation Trust, London, UK; 2 Division of Radiotherapy and Imaging, The Institute of Cancer Research, London, UK; 3 National Physical Laboratory, Teddington, UK

**Keywords:** Quality control, Quality assurance, Healthcare, Artificial intelligence, Magnetic resonance imaging, Image reconstruction

## Abstract

**Objective:**

AI-based MRI reconstruction techniques improve efficiency by reducing acquisition times whilst maintaining or improving image quality. Recent recommendations from professional bodies suggest centres should perform quality assessments on AI tools. However, monitoring long-term performance presents challenges, due to model drift or system updates. Radiologist-based assessments are resource-intensive and may be subjective, highlighting the need for efficient quality control (QC) measures. This study explores using image quality metrics (IQMs) to assess AI-based reconstructions.

**Materials and methods:**

58 patients undergoing standard-of-care rectal MRI were imaged using AI-based and conventional T2-weighted sequences. Paired and unpaired IQMs were calculated. Sensitivity of IQMs to detect retrospective perturbations in AI-based reconstructions was assessed using control charts, and statistical comparisons between the four MR systems in the evaluation were performed. Two radiologists evaluated the image quality of the perturbed images, giving an indication of their clinical relevance.

**Results:**

Paired IQMs demonstrated sensitivity to changes in AI-reconstruction settings, identifying deviations outside ± 2 standard deviations of the reference dataset. Unpaired metrics showed less sensitivity. Paired IQMs showed no difference in performance between 1.5 T and 3 T systems (*p* > 0.99), whilst minor but significant (*p* < 0.0379) differences were noted for unpaired IQMs.

**Discussion:**

IQMs are effective for QC of AI-based MR reconstructions, offering resource-efficient alternatives to repeated radiologist evaluations. Future work should expand this to other imaging applications and assess additional measures.

**Supplementary Information:**

The online version contains supplementary material available at 10.1007/s10334-025-01253-3.

## Introduction

AI-based MRI reconstruction techniques can improve workflow efficiency and the patient experience by reducing acquisition time whilst maintaining or improving image quality [1–3]. However, it is crucial for clinical institutions to ensure that AI-based techniques provided by MRI manufacturers meet local clinical requirements [4]. Local assessments often rely on one-off qualitative studies that compare image quality between AI-based and standard techniques via radiological scoring [5–8]. Such studies provide important insights into the technique’s performance at implementation, as a form of acceptance test. However, these assessments are potentially subjective, resource-intensive and impractical for subsequent monitoring of performance that may change over time, because of model drift or changes associated with MR system updates.

Quality assurance (QA) programmes, which include objective quality control (QC) tests using test objects (phantoms), are well established for MR hardware [9–13]. QC tests are routinely conducted at acceptance of the device to establish a reference dataset, and for ongoing monitoring (constancy tests) [11]. However, it is not yet known whether conventional phantom-based measurements are appropriate tools for assessing AI-based reconstruction methods trained on human data. The sensitivity of AI-based reconstructions to hardware degradation is also unknown.

As AI-based MR reconstruction techniques are increasingly adopted, professional bodies recommend developing frameworks concerning the implementation of these techniques into clinical practice [14–19]. It is often recommended that local institutions perform ongoing monitoring of AI-based tools in MRI, or radiology more generally, due to known issues around performance drift [20], sensitivity to variability in the input data, robustness to edge cases, training data bias [21], and hallucinations [22]. However, there is little literature describing practical methods that would be applicable for inclusion in an ongoing QA programme [22, 23]. There is an unmet need for efficient and effective QC performance measures of AI-based reconstruction techniques applied to MR imaging. Given the growing recommendations to implement QA for AI-based techniques in radiology, it is also essential to more thoroughly consider the associated resource demands on clinical institutions.

An effective QA programme in radiology includes establishing a framework that defines the quality objectives, performance measures, baseline performance, monitoring frequencies, feedback mechanisms, and accountability mechanisms [11, 12, 24]. Quality assessments for AI-technology should also fit into the well-defined requirements for managing medical imaging devices [18], including an acceptance test followed by longitudinal constancy tests. Any tests of AI-based MR reconstruction techniques should, therefore, be complementary to well-established hardware QA programmes. Appropriate QC performance measures should be quantifiable, objective, reproducible, generalisable across multiple systems and applications, simple to measure and interpret, and sensitive to changes in performance whilst being robust against inconsequential factors [11].

Objective image quality metrics (IQMs) are commonly used to optimise and evaluate the performance of image-based AI techniques [25–27]. They are also commonly used in the field of AI-based MR reconstructions [28–30]. IQMs enable users to mathematically compare the results of a reference image and a generated image, without resource-intensive and subjective user interpretation. By computing multiple complementary IQMs, they also allow for the investigation of different aspects of image quality simultaneously, from signal-to-noise ratio (SNR) to structural and textural properties [29–32]. Investigation into the relationship of IQMs to radiologist scores has also been explored [33]. Paired (full-reference) metrics offer a direct mathematical comparison between the AI-based images and the standard images and are commonly used for image quality assessments that have a “ground truth”. Unpaired (no-reference) metrics can be used in scenarios where a reference image is unavailable and attempt to correlate with human perception of quality [25].

Primary rectal cancer staging is performed using MRI and is underpinned by the use of multiple high-resolution two-dimensional small field of view (SFOV) turbo spin echo (TSE) T2-weighted (T2w) acquisitions [34, 35], each typically taking around 5 min to complete. With the use of AI-based reconstructions, we have previously shown that consequential acquisition time savings can be achieved without reducing image quality [36].

The aims of this study were to develop quantitative QC performance measures that enable longitudinal evaluation of AI-based reconstruction techniques in MRI to detect changes in performance, without the need for repeated resource-intensive assessments. We explored the suitability of commonly used IQMs to determine their utility as performance metrics and investigated their generalisability across different MR systems and different AI-based reconstruction settings. We applied these methods in rectal cancer imaging at our institution.

## Materials and methods

This was a prospective, single-centre study approved by a national research ethics committee (ClinicalTrials.gov Identifier: NCT05118555). Verbal consent was obtained from all patients for additional imaging as part of routine clinical examinations.

### Participants

**Fig. 1:**
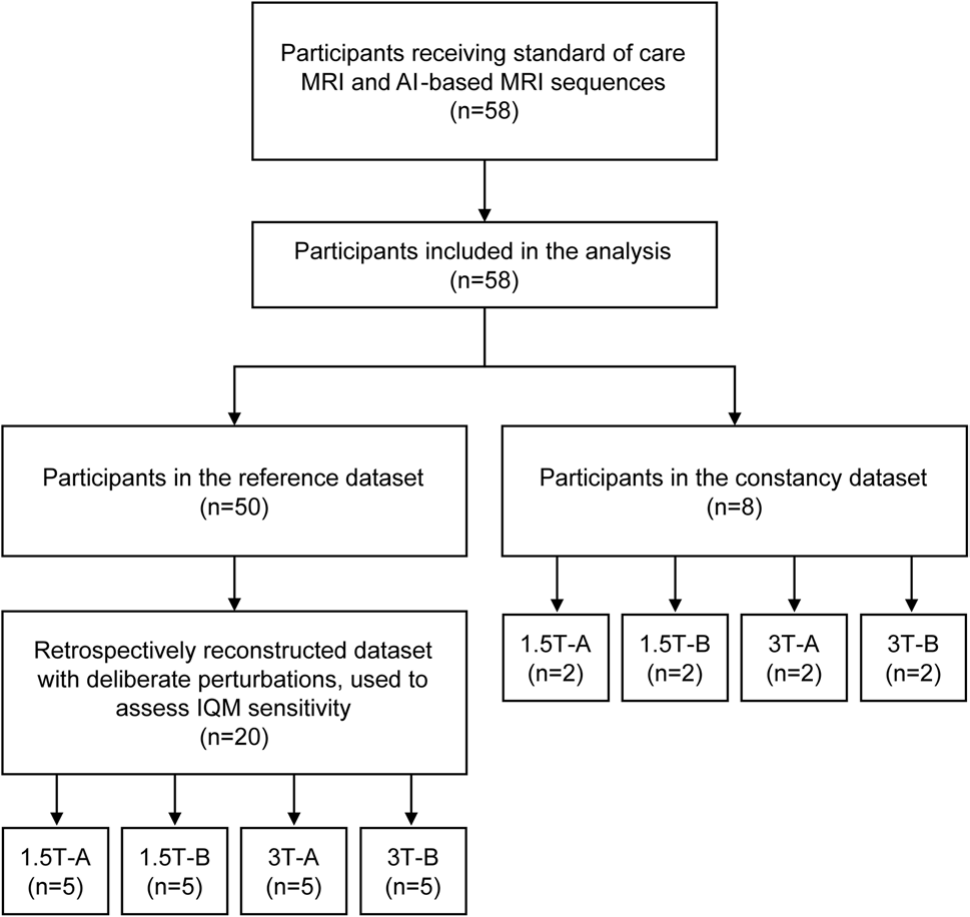
Study consort diagram

58 patients were recruited for enrolment in the study, as shown in Fig. 1. Potentially eligible patients were identified from those undergoing routine standard of care anorectal MRI between April 2023 and August 2024. Adult patients were screened for those who still had rectum in situ, typically those with: i) anorectal cancer (e.g. squamous cell carcinoma or adenocarcinoma) who had a complete clinical response and were on a “watch and wait” pathway, ii) a new diagnosis of cancer, or iii) receiving neo-adjuvant therapy prior to surgery. Patients who had previous abdominoperineal excision of rectum (APER) were not eligible for inclusion; however, patients who had undergone surgery (e.g. anterior resection) with sufficient residual rectum to enable rectal wall assessment were eligible. Patients undergoing standard of care imaging on a scanner without AI-based reconstruction capability were excluded.

The values of the IQMs for the first 50 patient exams form the reference dataset, against which further constancy tests and perturbation datasets were compared. This reference dataset has associated radiologist image quality scores [36]. A further eight patients formed part of the constancy test dataset.

### Data acquisition

Patients underwent a single MR examination on either 1.5 T MAGNETOM Sola (*n* = 26) or 3 T MAGNETOM Vida (*n* = 32) scanners (Siemens Healthineers, Erlangen, Germany) using a 30-channel anterior body array and 32-channel posterior spine array. Four MR systems were included in the evaluation, labelled as 1.5 T-A (*n* = 12), 1.5 T-B (*n* = 14), 3 T-A (*n* = 26), and 3 T-B (*n* = 6), respectively. AI-based reconstruction [37] (Deep Resolve, Siemens Healthineers, Erlangen, Germany) was applied in accelerated SFOV axial T2w-TSE sequences for denoising (DR-Boost) and super-resolution (DR-Sharp), that were acquired in addition to standard-of-care T2w-TSE imaging at matched slice positions. Participants were numbered consecutively in the study, irrespective of scanner. Participant motion was reduced as much as reasonably possible during the exam, as required for routine clinical scanning. Immediately before the acquisition, participants without contraindications were given an intramuscular injection of 20 mg hyoscine butylbromide antispasmodic or 1 mg Glucagon. Usual steps were taken to immobilise the participant comfortably on the couch, as per standard clinical practice. Participants were instructed to remain as still as possible during the acquisition, and the pair of AI-based and standard series were acquired consecutively during the exam in matched slice positions. This study also uses imaging of the pelvis, which is less affected by motion than abdominal or thoracic imaging. No significant MR system updates, to hardware or software, were installed during the study period.

The image data (digital imaging and communications in medicine, DICOM, format) and raw data were saved immediately after the MR examination, ensuring that the data pertaining to the original baseline performance were recorded.

The acquisition parameters are shown in Table 1, as described previously [36].

**Table 1:** Acquisition parameters

Parameter		AI-based	Standard
Acquisition time (min:s) (range)		01:28–02:37	04:02–08:24
Reconstructed voxel size (mm^3^)		0.3 × 0.3 × 3.0	0.3 × 0.3 × 3.0
Slices (range)		21–33	21–33
Averages		2	4
Parallel imaging factor		4	2
Phase oversampling		188%	100%
Refocussing flip angle (range)		160°	134°-160°
TR (ms) (range)	1.5 T: 3 T:	4650–5410 5240–8240	4650–5410 5240–8240
TE (ms)	1.5 T: 3 T:	87 96	87 96
Turbo factor	1.5 T: 3 T:	17 25	17 25
Other		Deep resolve boost (denoising) and sharp (super-resolution)	Zero-filling interpolation

TR: Repetition time, TE: Echo time. Minor flip angle variation was required for the standard T2-weighted TSE sequence for some participants to stay in Normal Mode of operation for radiofrequency exposure, but this did not have a substantial effect on the image contrast [36]

### Calculation of image quality metrics

**Fig. 2:**
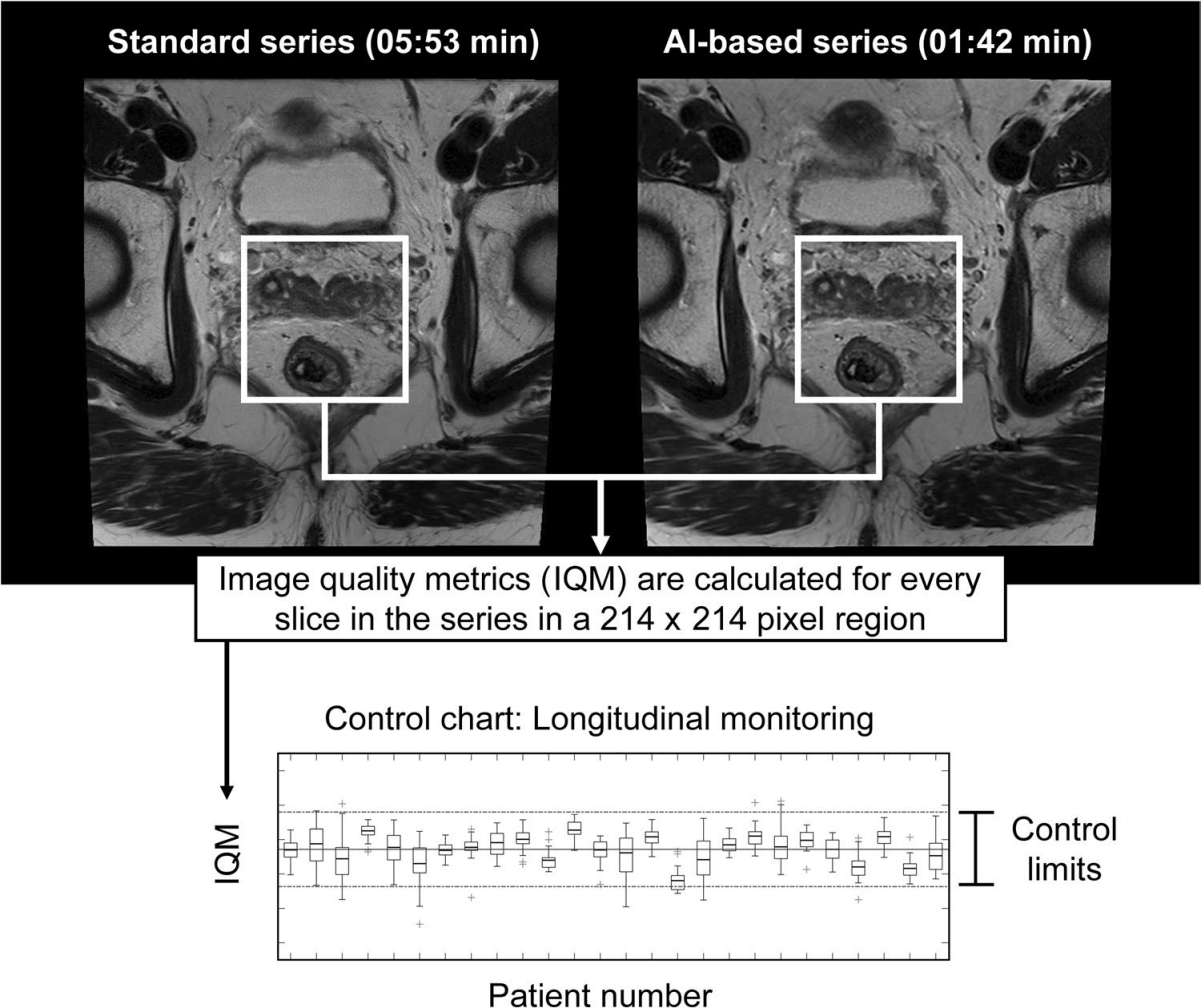
Diagram showing the overall image quality metric (IQM) calculation process. IQMs are calculated from a 214-by-214 pixel region in the centre of each image (640-by-640 pixels). Paired metrics give a single value, whilst unpaired metrics are computed for both the AI-based and standard series. An IQM is calculated from every slice in the series for that patient, which is then displayed in a control chart. Box plots displaying the mean, inter-quartile range, minima, maxima, and outliers were calculated for each patient

A range of IQMs were measured in MATLAB (version 9.9, R2020b, Natick, Massachusetts: The MathWorks Inc.) [38] to assess differences between the images from the AI-based reconstruction and standard images. Paired (full-reference) and unpaired (no-reference) metrics were investigated. Paired metrics were the mean squared error (MSE) [38], peak signal-to-noise ratio (pSNR) [38], structural similarity index (SSIM) [38, 39], visual information fidelity (VIF) [40], spectral angle mapper (SAM) [41], Bhattacharyya distance (BD) [42, 43], Bhattacharyya coefficient (BC) [42, 43], Chi-square distance (*χ*^2^D) [44], Frobenius norm (FN) [45], weighted Jaccard similarity (WJS) [46], and Bray–Curtis dissimilarity (BCD) [47]. Unpaired metrics were textural features (image entropy) [38], Blind/Referenceless Image Spatial Quality Evaluator (BRISQUE) [48], and Tenengrad [49]. Paired metrics give a single value for each pair of AI-based and standard imaging slices, using the standard sequence as the reference image, whilst unpaired metrics must be calculated for the AI-based and standard series separately. The natural log of the IQMs was taken in an attempt to ensure the data were normally distributed and to improve control chart visualisation [50, 51]. All metrics were calculated for a central 214 × 214-pixel region of interest (ROI) for every slice in the series, which is one-third of the original image dimensions in the x- and y-directions, as shown in Fig 2. This ROI was chosen as it contains the most relevant anatomical information, reduces the computational burden, and reduces the effect of inter-series motion on the IQM values. Definitions of all the metrics used in the evaluation are available in the Supplementary Material, with selected metric definitions provided in Table 2.

**Table 2:** Definitions of selected image quality metrics. Metrics are used to assess an image ($I$) with a total of $N$ pixels in an $m$ by $n$ array

Metric	Definition	References
Peak signal-to-noise ratio (pSNR)	${\text{pSNR}}\left(I,\widehat{I}\right)=10{\text{log}}_{10}\frac{{{\text{max}}\left(\widehat{I}\right)}^{2}}{{\text{MSE}}\left(I,\widehat{I}\right)}$ Where the mean squared error (MSE) is, ${\text{MSE}}\left(I,\widehat{I} \right)= \frac{1}{N}{\sum }_{i=1}^{m}{\sum }_{j=1}^{n}{\left({I}_{ij}-{\widehat{I}}_{ij}\right)}^{2}$	[30, 38]
Structural similarity index (SSIM)	${\text{SSIM}}\left(I,\widehat{I}\right)=\frac{\left({2\mu }_{I}{\mu }_{\widehat{I}}+{c}_{1}\right)\left({2\sigma }_{I,\widehat{I}}+{c}_{2}\right)}{\left({\mu }_{I}^{2}+{\mu }_{\widehat{I}}^{2}+{c}_{1}\right)({\sigma }_{I}^{2}+{\sigma }_{\widehat{I}}^{2}+{c}_{2})}$ Where: ${\mu }_{I}$ is the mean of $I$ ${\sigma }_{I}^{2}$ is the variance of $I$ ${\sigma }_{I, \widehat{I}}$ is the covariance of $I$ and $\widehat{I}$ ${c}_{1}$ = ${\left({k}_{1}D\right)}^{2},{c}_{2}\text{ = }{\left({k}_{2}D\right)}^{2}$ where, ${k}_{1}$ is 0.01 and ${k}_{2}$ is 0.03 by default $D$ is the dynamic range	[38, 39]
Visual information fidelity (VIF)	Please refer to the original publication due to complexity	[40]
Spectral angle mapper (SAM)	${\text{SAM}}\left(I,\widehat{I}\right)= {\text{cos}}^{-1}\left(\frac{I \cdot \widehat{I}}{\Vert I\Vert \cdot \Vert \widehat{I}\Vert }\right)$ Here, each 2D image ($I$ and $\widehat{I}$) have been reshaped into a 1D vector of pixel intensities	[41]
Image entropy	$\text{Entropy(}I\text{)}=-{\sum }_{k=1}^{D}{p}_{k}(I){\text{log}}_{2}({p}_{k}(I))$ Here, ${p}_{k}(I)=\frac{{h}_{k}(I)}{N}$, where​ ${h}_{k}(I)$ is the count of pixels in image $I$ at intensity $k$, $N$ is the total number of pixels in the image, and $D$ is the dynamic range	[38]
Blind/referenceless image spatial quality evaluator (BRISQUE)	Please refer to the original publication due to complexity	[38, 48]
Tenengrad	$\text{Tenengrad(}I\text{)}=\frac{1}{N}{\sum }_{i=1}^{m}{\sum }_{j=1}^{n}\left({\nabla }_{x}{I}_{ij}^{2}+{\nabla }_{y}{I}_{ij}^{2}\right)$ Here, ${\nabla }_{(x,y)}$ is the gradient in the $x$- or $y$-direction	[30, 49]

For full-reference metrics, $I$ is compared to a reference image $\left(\widehat{I}\right)$ of the same dimensions. Here, ${I}_{i,j}$ and ${\widehat{I}}_{i,j}$ are corresponding pixels of the test and reference image. See the Supplementary Material 6 for definitions of all the metrics used in the evaluation

### Sensitivity to perturbations

Sensitivity of the IQMs to changes in the reconstruction process was evaluated using five patient datasets from each MR system, which were retrospectively reconstructed (RR) from the raw k-space data with deliberate perturbations to the reconstruction process. These perturbations were chosen to reflect plausible changes that could occur due to an update in the AI-based reconstruction method. All modifications were performed using the MR system reconstruction tools provided by the manufacturer. The following perturbations were applied; RR1) an increase in the AI-reconstruction mode denoising strength from “medium” to “high”, RR2) a decrease in the AI-reconstruction mode denoising strength from “medium” to “low”, and RR3) an additional image edge enhancement and smoothing filter.

### Image quality scoring of perturbations

Qualitative image scoring of the perturbed datasets was performed by two subspecialist radiologists, with experience in rectal cancer imaging of 11 and 7 years respectively, to indicate whether the perturbations were of clinical relevance. The radiologists visually assessed the perturbed images and scored them using a Likert scale across four different qualitative image features. The features were chosen to match those required to accurately stage tumours, as in previous work [36]: signal-to-noise ratio (SNR) (qualitative visual assessment of how the rectal wall signal compares to background noise with score 1 referring to excessive noise levels such that the rectal wall anatomy cannot be reliably assessed, and score 4 such that image noise is minimal and not impacting on the assessment), rectal wall sharpness (the level of sharpness or blurriness of the anatomy), rectal wall layer conspicuity (ability to define the constituent rectal wall layers including mucosa, submucosa and muscle layer) and overall image quality. Each image quality feature was scored using the following scale: 1 = unacceptable/non-diagnostic, 2 = adequate, 3 = good, 4 = excellent. Radiologists were also asked to comment on the presence of any image artefacts. The perturbed images were displayed side-by-side in a random order, and without any annotations, using the institutional picture archiving and communication system (Sectra IDS7 PACS system version 24.1, Sectra AB, Linköping, Sweden).

### Statistics and control charts

Statistical calculations were performed in the R software environment for statistical computing and graphics [52], and using G*Power3.1 [53]. Control charts were plotted using MATLAB.

The values of the IQMs for the reference, constancy, and perturbation datasets were evaluated using a Shewhart control chart [54, 55]. The control chart statistical tests compared the mean value of the IQM for each patient examination against the mean and standard deviation (SD) of the reference dataset. Control chart tests are displayed in Table 3. Limits were chosen according to standard control chart methods [55]. A value within ± 2 SD of the reference measures would be considered acceptable. A value outside these limits (Rule 1) triggers additional investigation; (Rules 2–4) are applied sequentially to the IQM values with the failure of any rule leading to further investigation.

**Table 3:** Control chart rules

Rule	Description
1	Warning: The mean value of the QC test for the patient examination exceeds the control limit set as the mean ± 2 SD of the reference dataset. Trigger rules 2–4 sequentially until a control limit is exceeded
2	3-SD rule: The mean value of the QC test for the patient examination exceeded the control limit set as the mean ± 3 SD of the reference dataset. Trigger additional inspection of the data
3	Twice 2-SD rule: The mean value of the QC test for the patient examination has exceeded the control limit set as the mean ± 2 SD of the reference dataset for two consecutive tests. Trigger additional inspection of the data
4	Four 1-SD rule: The mean value of the QC test for the patient examination exceeded the control limit set as the mean ± 1 SD of the reference dataset for four consecutive tests. Trigger additional inspection of the data

QC: quality control, SD: standard deviation

Metrics were deemed to be sensitive to perturbations in the reconstruction if any of the Rules 2–4 in Table 3 were activated.

Linear mixed-effects models were used to assess the differences between the four MR systems for each IQM using the *lme4*-package [56], where the IQM was the response and MR system was the fixed effect. Participants were included as a random effect on the intercept. A post hoc comparison of the estimated marginal means from the mixed-effects models for each MR system was conducted using the *emmeans*-package [57].

Differences between the median Likert scores for the perturbed images (*n* = 20) and the original AI-based axial images (*n* = 50) [36] were evaluated using a Mann–Whitney *U* test. The threshold for statistical significance of Benjamini–Hochberg corrected p-values was *p* = 0.05.

## Results

### Patient characteristics

Demographic details are outlined in Supplementary Material 1. 58 participants were enrolled (median age 62.5 years, range 33–89 years, 31 male). The majority (57%) of patients were treated for adenocarcinoma. 64% had imaging as part of an active surveillance “watch and wait” pathway and the remainder for staging and response assessment. The majority of tumours were present in the anus or lower rectum (34% and 33%, respectively).

### IQM control charts

**Fig. 3:**
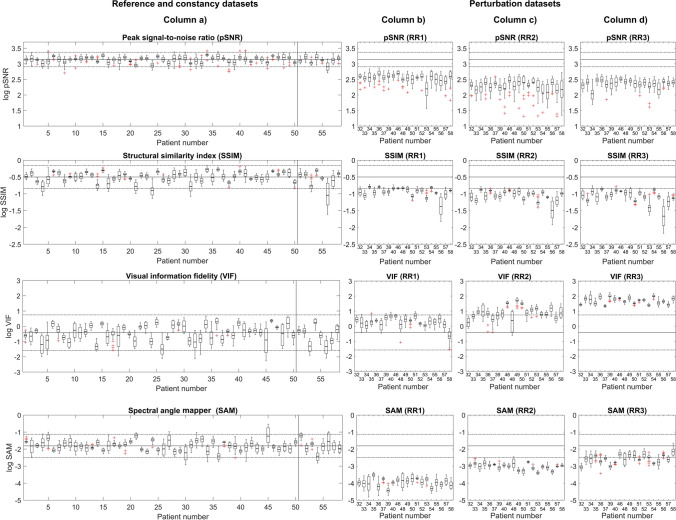
Paired (full-reference) image quality metrics (IQMs) are plotted for the reference and constancy datasets (column **a**) and perturbation datasets (columns **b**–**d**). Box plots displaying the mean, inter-quartile range, minima, maxima, and outliers were calculated for each individual patient and show the value of the IQMs comparing the images with and without AI-based reconstructions. A selection of paired IQMs are shown: peak signal-to-noise ratio (pSNR), structural similarity index (SSIM), visual information fidelity (VIF), and spectral angle mapper (SAM). Solid horizontal lines indicate the mean IQM value for each reference dataset, whilst dashed lines indicate ± 2 standard deviations. The vertical solid line in columns **a** indicate the extent of the *n* = 50 patient reference dataset. The eight patients to the right of this line form the constancy dataset

The results of the reported IQMs calculated between the AI-based and standard series for all MR systems are shown in Figs. 3 (paired metrics) and 4 (unpaired metrics). For simplicity, Fig. 3 only displays a selection of IQMs (pSNR, SSIM, SAM, and VIF). The control chart results for other paired IQMs are provided in Supplementary Material 2.

**Fig. 4:**
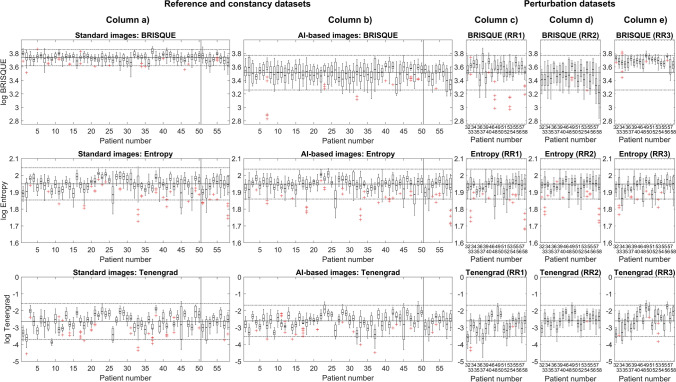
Unpaired (no-reference) image quality metrics (IQMs) are plotted for the reference and constancy datasets (columns **a** and **b**) and perturbation datasets (columns **c**–**e**). Box plots displaying the mean, interquartile range, minima, maxima, and outliers were calculated for each individual patient and show the value of the IQMs comparing the images with and without AI-based reconstructions. Three unpaired IQMs were calculated: textural features (entropy), blind/referenceless image spatial quality evaluator (BRISQUE), and Tenengrad. Solid horizontal lines indicate the mean IQM value for each reference dataset, whilst dashed lines indicate ± 2 standard deviations. The vertical solid line in columns **a** and **b** indicate the extent of the *n* = 50 patient reference dataset. The eight patients to the right of this line form the constancy dataset

In both Figs. 3 and 4, the reference and constancy datasets are displayed alongside the perturbation datasets, which gauge the sensitivity of each metric to the deliberate RR-modifications (RR1, RR2, and RR3).

### Constancy tests

Results of the paired metrics (pSNR, SSIM, SAM, and VIF) for the reference and constancy datasets are displayed in Fig. 3 (column a) and in Supplementary Material 2. These show no appreciable deviation in the constancy tests compared to the reference datasets, over the course of the study period. In Fig. 3, the ± 2SD control limit was exceeded for one patient in the constancy dataset for the SSIM metric, which was attributed to gross patient motion, rather than a system performance issue.

Figure 4 (columns a and b) show the results from the unpaired IQMs (BRISQUE, entropy, and Tenengrad) that were calculated for the reference and constancy datasets. These also show no appreciable deviation in the constancy tests compared to the reference datasets, over the course of the study period. The ± 2SD control limit was exceeded for one patient in the constancy dataset (standard images) for the Tenengrad metric.

### Sensitivity to perturbations

**Fig. 5:**
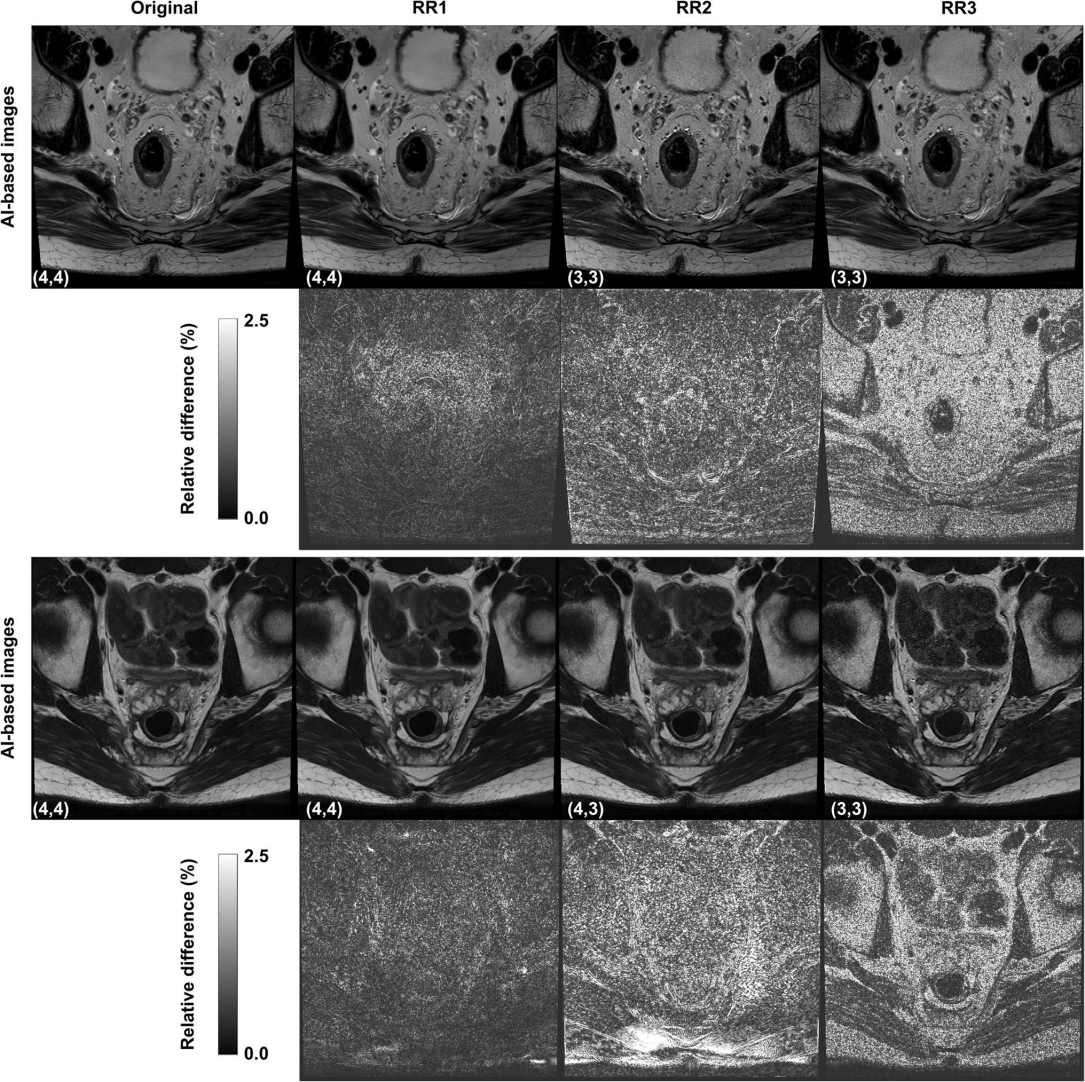
Example axial T2-weighted turbo spin echo (TSE) images acquired using the AI-based reconstruction technique for two patients. Top rows: a 56-year-old male patient treated with chemoradiotherapy for a T2-stage rectal cancer and subsequent complete clinical response, scanned at 3 T. Bottom rows: a 36-year-old male patient treated with chemoradiotherapy for T2-stage rectal cancer and subsequent good partial response, scanned at 1.5 T. Both patients were on a “watch and wait” active surveillance protocol. The original reconstruction and retrospective reconstruction (RR) images are shown. The RR reconstructions are calculated with deliberate perturbations: RR1) an increase in the AI-reconstruction mode denoising strength from “medium” to “high”, RR2) a decrease in the AI-reconstruction mode denoising strength from “medium” to “low”, and RR3) an additional image edge enhancement and smoothing filter. The Radiologist scores for overall image quality are displayed in the corners of the images (Radiologist 1, Radiologist 2). Note that these scores were measured per series, not per slice. Relative difference images calculated between the original images and RR images are shown. The relative difference images are calculated relative to the maximum intensity of the original image and displayed as a percentage, i.e. $\left|\text{Original image - Perturbed image}\right|/\text{max(Original image)}$

Example images showing the effect of the deliberate perturbations are shown in Fig. 5. The perturbation datasets in Fig. 3 (columns b, c, and d) and in Supplementary Material 2 show that all the paired IQMs have a degree of sensitivity to the perturbations, but to different extents. All the paired metrics would have triggered Rule 1 and at least one subsequent Rule (2–4), indicating they are sensitive to perturbations in the reconstruction pipeline.

The results for pSNR and SSIM indicate that these metrics have broadly similar responses to the different perturbations. The VIF metric demonstrated variable sensitivity, with no rules being triggered for RR1, but RR2 and RR3 triggering Rule 1 and at least one subsequent Rule (2–4), with RR3 having the largest effect magnitude. The SAM IQM also demonstrated variable sensitivity, with the mean value for every patient in the perturbed dataset being outside the ± 2SD control limit for RR1 and RR2, but less sensitivity to RR3, with 12/20 patients outside the ± 2SD control limit.

The results in Fig. 4 (columns c, d, and e) show that these unpaired IQMs are not as sensitive to perturbations in the reconstruction pipeline, compared to paired metrics in Fig. 3. The entropy values were not appreciably different for any of the datasets, with none of Rules 2–4 being triggered. The BRISQUE IQM was sensitive to changes due to RR3, although only Rule 4 (Four 1-SD) would have applied. The entropy of the standard and AI-based series is comparable and all within the control limit, whereas BRISQUE shows a difference between AI-based and standard series. The Tenengrad IQM similarly did not trigger any of Rules 2–4, and the AI-based and standard series are comparable.

### MR system comparison

The results of the linear mixed-effects models comparing the IQMs for the 58 patients across the four different MR systems are shown in Supplementary Material 5. The 3 T-A and 3 T-B scanners were not significantly different for any metric (*p* > 0.99), and the same was true for the 1.5 T-A and 1.5 T-B scanners (*p* > 0.62). Only the unpaired metric scores were significantly different between MR systems, and only for the standard imaging series. Specifically, the scores for the standard imaging were significantly different when comparing 3 T-A and 1.5 T-A scanners for entropy (*p* = 0.007) and BRISQUE (*p* = 0.0379), and when comparing 3 T-A and 1.5 T-B scanners for entropy (*p* = 0.0092) and Tenengrad (*p* = 0.007).

Whilst the null hypothesis was rejected for unpaired metrics when performing a pairwise comparison between MR systems, the magnitude of the difference was small. The mean values of the entropy IQM across every MR system were always within ± 2 SD of the mean value of the 3 T-A system, the scanner with the largest number of datapoints. The graphical results for MR system comparisons are shown in Supplementary Material 3 and 4.

### Image quality scoring of perturbations

Both radiologists reported a significant decrease in image quality for the RR2 and RR3 perturbed datasets compared to the original reconstructions for all image features (*p* < 0.05). The scores for the RR1 perturbation showed a significant increase in perceived signal-to-noise ratio (*p* < 0.04), but no significant change in overall image quality (*p* > 0.65). The results from the image quality assessment are shown in Table 4, and example images of the perturbations can be seen in Fig. 5.

**Table 4:** Results of the Likert image scoring assessment for the perturbation dataset (*n* = 20 patients) compared to the original AI-based axial images (*n* = 50)

Features:		Signal-to-noise ratio			Rectal wall sharpness			Rectal wall layer conspicuity			Overall image quality		
Image series		Mean	Median	*p*-value	Mean	Median	*p*-value	Mean	Median	*p*-value	Mean	Median	*p*-value
Reader 1	Original	3.78	4.0	–	3.68	4.0	–	3.70	4.0	–	3.70	4.0	–
	RR1	4.00	4.0	0.040*	3.60	4.0	0.699	3.40	3.0	0.020*	3.75	4.0	0.657
	RR2	2.95	3.0	< 0.001*	3.20	3.0	0.002*	3.20	3.0	< 0.001*	3.10	3.0	< 0.001*
	RR3	2.10	2.0	< 0.001*	2.55	3.0	< 0.001*	2.55	2.5	< 0.001*	2.40	2.0	< 0.001*
Reader 2	Original	3.42	3.0	–	3.56	4.0	–	3.34	3.0	–	3.46	4.0	–
	RR1	3.80	4.0	0.006*	3.20	3.0	0.035*	3.45	4.0	0.615	3.55	4.0	0.711
	RR2	2.65	3.0	< 0.001*	2.80	3.0	< 0.001*	2.95	3.0	0.032*	2.55	3.0	< 0.001*
	RR3	2.35	2.0	< 0.001*	2.65	3.0	< 0.001*	2.45	2.5	< 0.001*	2.35	2.0	< 0.001*

Scores for the perturbation dataset that are significantly different from the original image scores are marked with an asterisk (*). A Benjamini–Hochberg (BH) correction has been applied to the *p*-values, with a significance threshold of *p* = 0.05

## Discussion

This study has assessed possible quantitative methods of evaluating MR images involving AI-based reconstruction techniques using IQMs, which constitute part of an ongoing QA programme.

For ongoing monitoring, we propose that a subset of patients receive both standard and AI-based axial T2-weighted TSE sequences. For instance, at our institution, we acquire a standard axial acquisition in the same position as the AI-based sequence for the first patient that meets the reference dataset inclusion criteria per scanner per month. The additional “monitoring” acquisition consists of one standard axial sequence matched to the accelerated sequence, adding approximately 6 min to the exam time. This duration is substantially less than the overall time-saving benefits of AI-based imaging (~ 20 min per exam), as routine rectum exams contain multiple high-resolution T2-weighted TSE acquisitions in different locations [34–36]. Whilst our study does not prescribe specific patient selection methods for other institutions, we recognise that these logistics will depend on local requirements, patient volumes, and the specific details of the AI-based imaging protocol.

This study aimed to develop and evaluate the use of quantitative IQMs for the longitudinal QC of AI-based MRI reconstruction techniques, specifically in the context of rectal cancer imaging. Through the evaluation of various paired and unpaired IQMs, we demonstrated that these metrics could serve as effective and resource-efficient tools for detecting changes in reconstruction performance over time, without the need for repeated, labour-intensive radiologist assessments.

Our findings show that all the paired IQMs, including pSNR, SSIM, VIF, and SAM, are sensitive to perturbations in the reconstruction process. These metrics responded to changes such as adjustments to denoising strength and the addition of image filters, providing evidence that these IQMs are suitable for detecting changes in image quality. In contrast, the unpaired metrics displayed lower sensitivity, particularly for the entropy and Tenengrad IQMs, suggesting that paired metrics may be better suited for AI-reconstructed MR image evaluation. As we are evaluating different reconstruction methods and have a ‘ground truth’ baseline, paired metric evaluation is an obvious choice to test performance.

The results of the linear mixed-effects models showed no significant differences could be detected between MR systems of the same field strength for any paired or unpaired metrics. Some minor differences were noted for the unpaired IQMs between 1.5 T and 3 T systems. Although these differences were statistically significant, they were small in magnitude, falling within ± 2 SD of the mean values from the reference dataset. This suggests that whilst there may be inherent variations between MR systems between 1.5 T and 3 T, as would be expected, the impact on IQMs is not clinically relevant. Our previous work did not detect any statistically significant difference in radiologist-perceived image quality between 1.5 T and 3 T datasets [36].

**Fig. 6:**
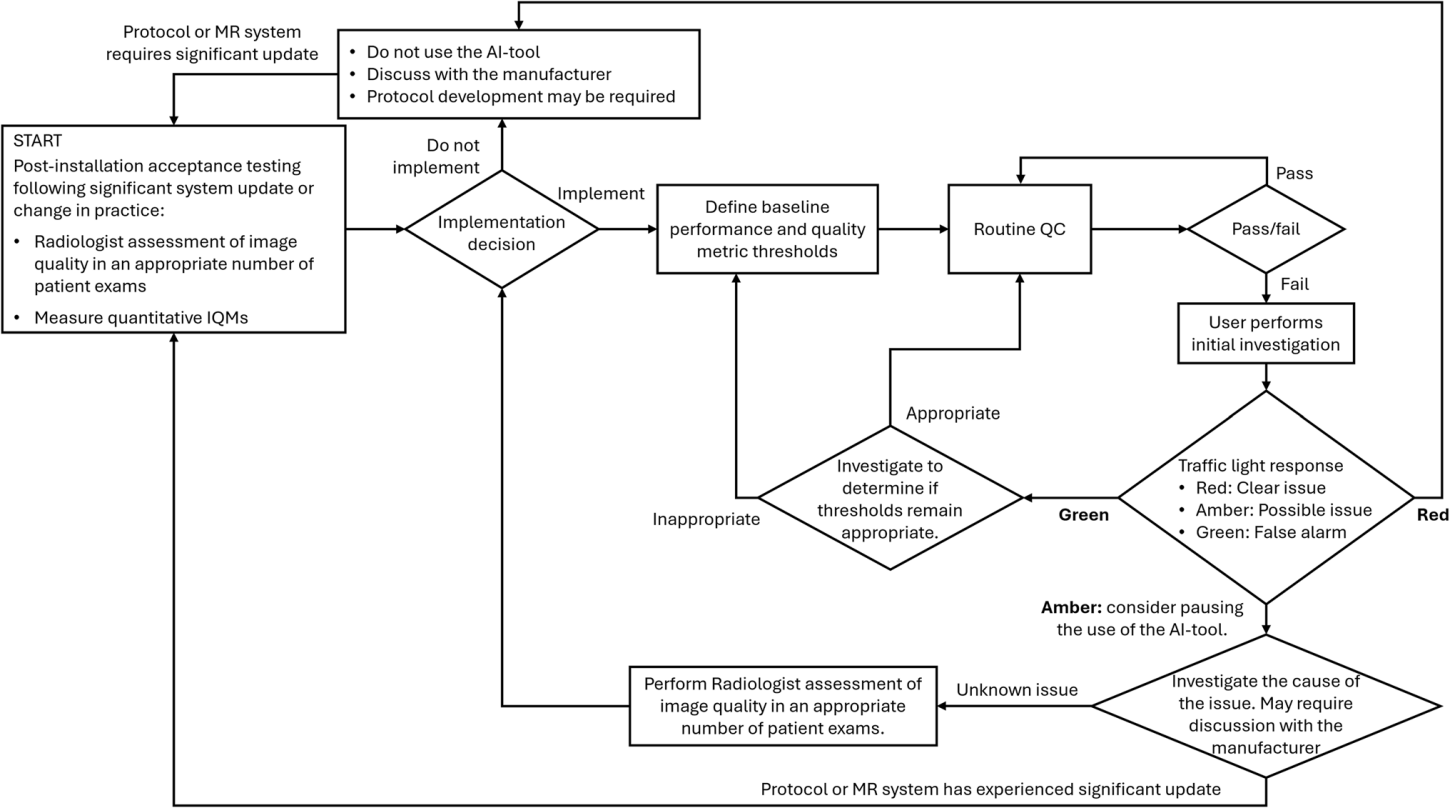
Example quality assurance workflow that would integrate an acceptance test, as performed in previous work [36], alongside longitudinal routine QC using quantitative IQMs

This study also shows that it is possible to establish baseline control limits for each IQM, which can be used for ongoing monitoring as part of a QA framework. A practical traffic light system, whereby green indicates acceptable performance, yellow signals a deviation requiring closer monitoring, and red suggests the need for immediate investigation, could provide a structured and clear QC strategy, for example as shown in Fig. 6. When an IQM exceeds ± 2 SD from the baseline, further investigation could be triggered. These control limits could provide an objective framework for QC assessments, offering an early warning system for performance drift, which may arise from changes in AI-reconstruction models or hardware updates. The importance of collecting an appropriate reference dataset is clearly paramount. The baseline data collection for the IQMs can occur alongside a radiologist image quality evaluation, as described in previous work [36].

The integration of these IQMs into an institutional QA framework requires careful planning, including defining when and how radiologist intervention is required when deviations in image quality are identified. As there is no standardised way of quantifying image quality, we aimed to put forward several metrics that were simple to calculate in commonly available software, which are sensitive to a range of image features. However, heterogeneity in metric selection remains a key challenge, and there are several calls for further work towards a standardised approach [22, 30].

Radiologist evaluations of image quality remain crucial when introducing new imaging techniques. This work is not intended to supersede these evaluations, which are important at the acceptance phase, but to act as complementary tests for ongoing monitoring. It is important to note that IQMs are only detecting differences between AI-based and standard images, without a comment on whether this change is an improvement or a degradation in quality. The Likert scoring assessment of the perturbed images show that the changes made to the reconstruction pipeline had a significant impact on the perceived image quality, indicating that they would be of clinical importance. Whilst some metrics presented here are able to detect clinically important perturbations, at this stage, the results are not specific enough to pinpoint precisely what kind of change has occurred, for example hallucinations that may mimic anatomical structures. Although no hallucinations were detected by the radiologists during the qualitative image scoring, detection of hallucinatory features has been described as a particular area of concern more widely and a topic that requires further development [22]. This is further incentive to include radiologist evaluations in an assessment framework. Whilst other work has investigated the correlation between IQMs and radiologist scoring [33], there is still a need to perform radiologist evaluations during acceptance of new AI techniques. Further work should explore the threshold at which changes in IQMs equate to a radiologist’s perception of clinically significant changes in the image that may preclude or affect diagnostic use. We will also consider whole image evaluation and on-line computation of these metrics.

This study has several limitations, including a relatively small sample size. This was a single-centre study conducted using MR systems from a single manufacturer which may limit generalisability to other sites and systems; however, previous work has included supplemental material to aid implementation more widely [36]. This study focussed on a limited number of IQMs, which is by no means exhaustive. Future work could consider additional commonly used metrics, but also their sensitivity to a range of different image perturbations and the appropriate frequency for constancy tests. The methodology presented here could be expanded to other body regions and MR pulse sequences, particularly those where AI-based reconstruction techniques are increasingly being adopted.

## Conclusion

Quantitative IQMs offer a promising method for resource-efficient QC of AI-based MR reconstructions. Our study demonstrated that paired IQMs are highly sensitive to changes in image reconstruction performance and can be employed to monitor image quality over time. We propose that these IQMs have the potential to be used as complementary tools alongside traditional radiologist-based assessments, forming an integral part of a broader QA program. Whilst there is growing guidance on the importance of assessing the performance of AI-based tools in clinical practice, there are currently limited established methods for doing so. This study represents an initial step in addressing this broader issue by proposing tests that can fit into well-established frameworks for longitudinal quality assurance. Future studies should aim to refine these metrics and extend their application to other AI-accelerated imaging applications. Further work will also assess the resources required for these QA programmes.

## Supplementary Information

Below is the link to the electronic supplementary material.

Supplementary file1 (PDF 1530 KB)

## Data Availability

The data that support the findings of this study are not openly available due to reasons of sensitivity and are available from the corresponding author upon reasonable request. Data are located in controlled access data storage at The Royal Marsden Hospital and the Institute of Cancer Research.
