# Supplementary material for: Quantitative image quality metrics enable resource-efficient quality control of clinically applied AI-based reconstructions in MRI
Source: MAGMA. 2025 May 24;38(3):547–60. doi: 10.1007/s10334-025-01253-3 (PMC12255595; doi:10.1007/s10334-025-01253-3)
Supplement: Supplementary file 1 — Supplementary file1 (PDF 1530 KB) [file 10334_2025_1253_MOESM1_ESM.pdf]

Supplementary material

Quantitative image quality metrics enable resource-efficient quality control of clinically-applied AI-based reconstructions in MRI

Journal:

Magnetic Resonance Materials in Physics, Biology and Medicine

Authors:

Owen A. White*[1,2]
Joshua Shur[1]
Francesca Castagnoli[1,2]
Geoff Charles-Edwards[1,2]
Brandon Whitcher[1,2]
David J. Collins[1,2]
Matthew T.D. Cashmore[3]
Matt G. Hall[3]
Spencer A. Thomas[3]
Andrew Thompson[3]
Ciara A. Harrison[1,2]
Georgina Hopkinson[1]
Dow-Mu Koh[1,2]
Jessica M. Winfield[1,2]

1) MRI Unit, The Royal Marsden NHS Foundation Trust, London, UK
2) Division of Radiotherapy and Imaging, The Institute of Cancer Research, London, UK
3) National Physical Laboratory, Teddington, UK

*Corresponding author: Owen.White@rmh.nhs.uk

Supplementary material content:
- 3 Figures
- 3 Tables
- 1 zip file containing MATLAB scripts

Supplementary material 1: Table of participant demographics. W+W: Watch and wait.

**Patient descriptor**	**Value**
Age (years) median, range	62.5, 33-89
Male sex (%)	31 (53%)
**Tumor**	
Squamous cell carcinoma	22 (38%)
Adenocarcinoma	33 (57%)
Other	3 (5%)
**Indication**	
Surveillance/W+W	37 (64%)
Staging/Response assessment	21 (46%)
Prior radiotherapy	51 (87%)
**Surgery**	
None	49 (84%)
Anterior resection	4 (7%)
Local excision	5 (9%)
**Location**	
Anus	20 (34%)
Low rectum	19 (33%)
Mid rectum	13 (22%)
Upper rectum	4 (7%)
Sigmoid colon	2 (3%)
**T-Stage (SCC)**	
T1	2 (9%)
T2	10 (45%)
T3	6 (28%)
T4	4 (18%)
**T-Stage (Adenocarcinoma)**	
T1	5 (15%)
T2	5 (15%)
T3a	2 (6%)
T3b	7 (21%)
T3c	7 (21%)
T3d	2 (6%)
T4a	0 (0%)
T4b	5 (15%)

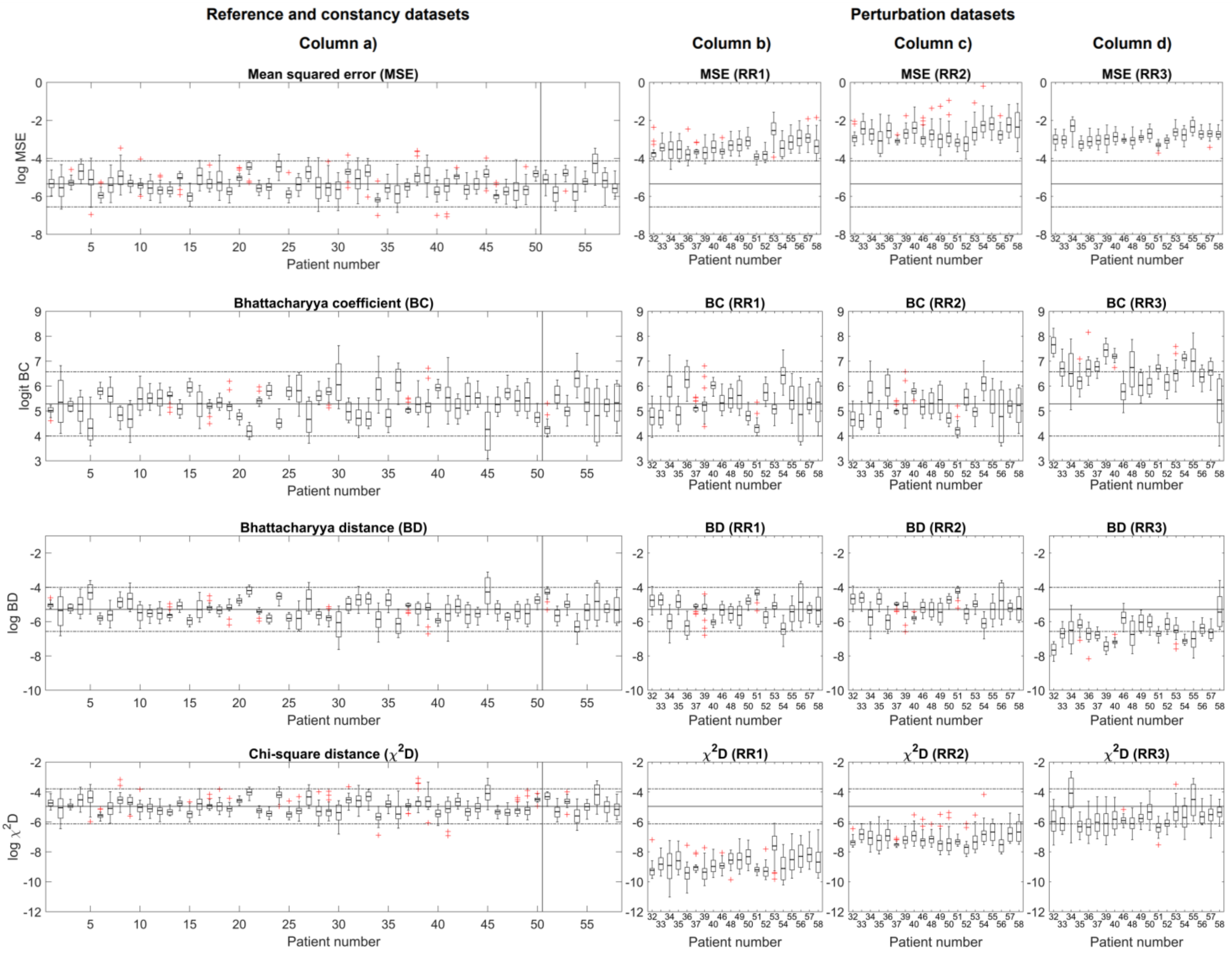

Supplementary material 2: Control charts for IQMs not shown in the main text.

Paired (full-reference) image quality metrics (IQMs) are plotted for the reference and constancy datasets (column a) and perturbation datasets (columns b-d). Box plots displaying the mean, inter quartile range, minima, maxima, and outliers were calculated for each individual patient and show the value of the IQMs comparing the axial images with and without AI-based reconstructions. Paired IQMs that were not shown in the main text are displayed: Mean-squared error (MSE), Bhattacharyya coefficient (BC), Bhattacharyya distance (BD), and Chi-square distance (χ^2D). Note that a logit transform was applied to BC. Solid horizontal lines indicate the mean IQM value for each reference dataset, while dashed lines indicate ±2 standard deviations. The vertical solid line in column a) indicates the extent of the n=50 patient reference dataset. The eight patients to the right of this line form the constancy dataset. (*Figure continued overleaf*)

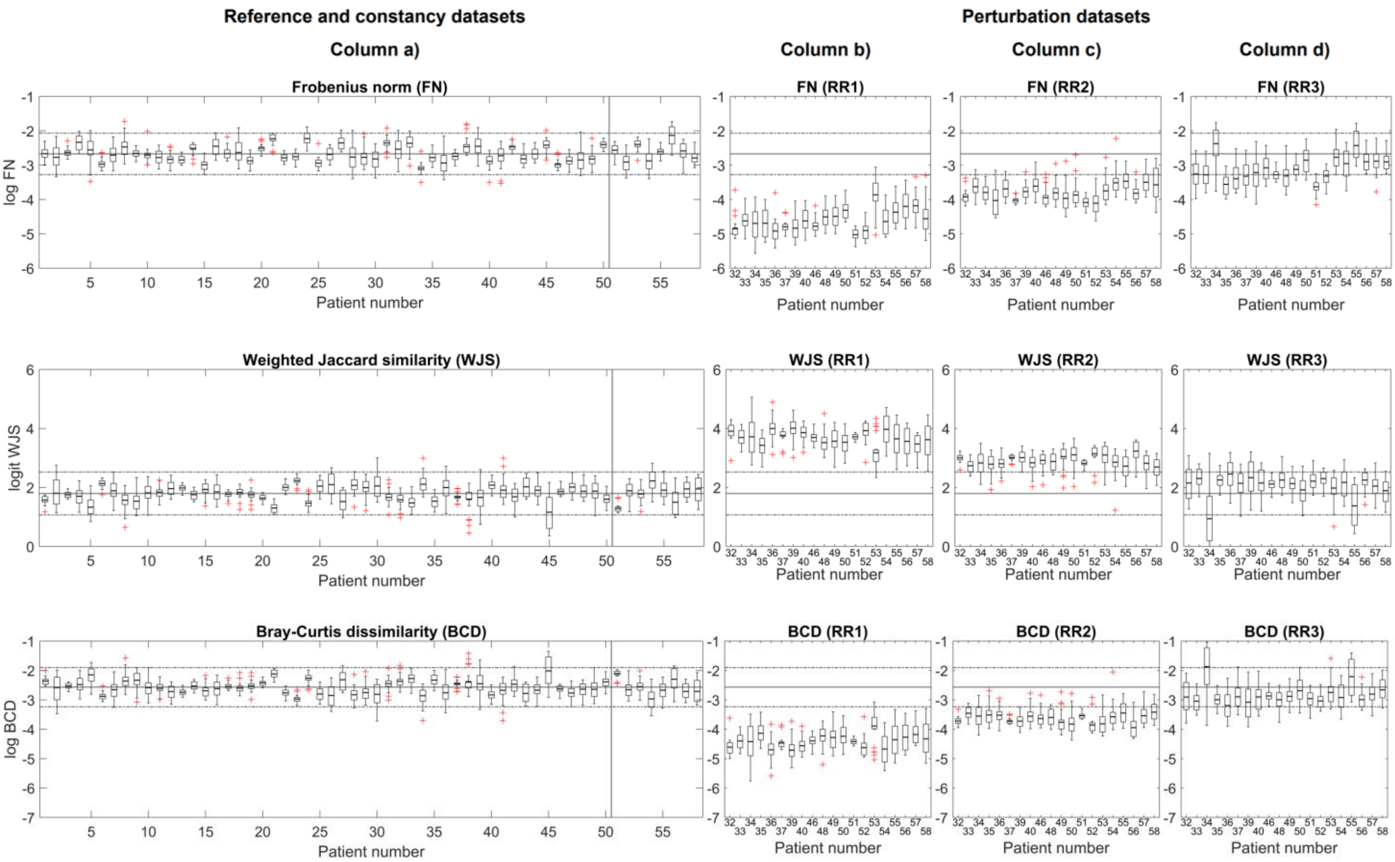

Supplementary material 2: (*continued*) Control charts for IQMs not shown in the main text. Paired (full-reference) image quality metrics (IQMs) are plotted for the reference and constancy datasets (column a) and perturbation datasets (columns b-d). Box plots displaying the mean, inter quartile range, minima, maxima, and outliers were calculated for each individual patient and show the value of the IQMs comparing the axial images with and without AI-based reconstructions. Paired IQMs that were not shown in the main text are displayed: Frobenius norm (FN), Weighted Jaccard similarity (WJS), and Bray-Curtis dissimilarity (BCD). Note that a logit transform was applied to WJS. Solid horizontal lines indicate the mean IQM value for each reference dataset, while dashed lines indicate ±2 standard deviations. The vertical solid line in column a) indicates the extent of the n=50 patient reference dataset. The eight patients to the right of this line form the constancy dataset.

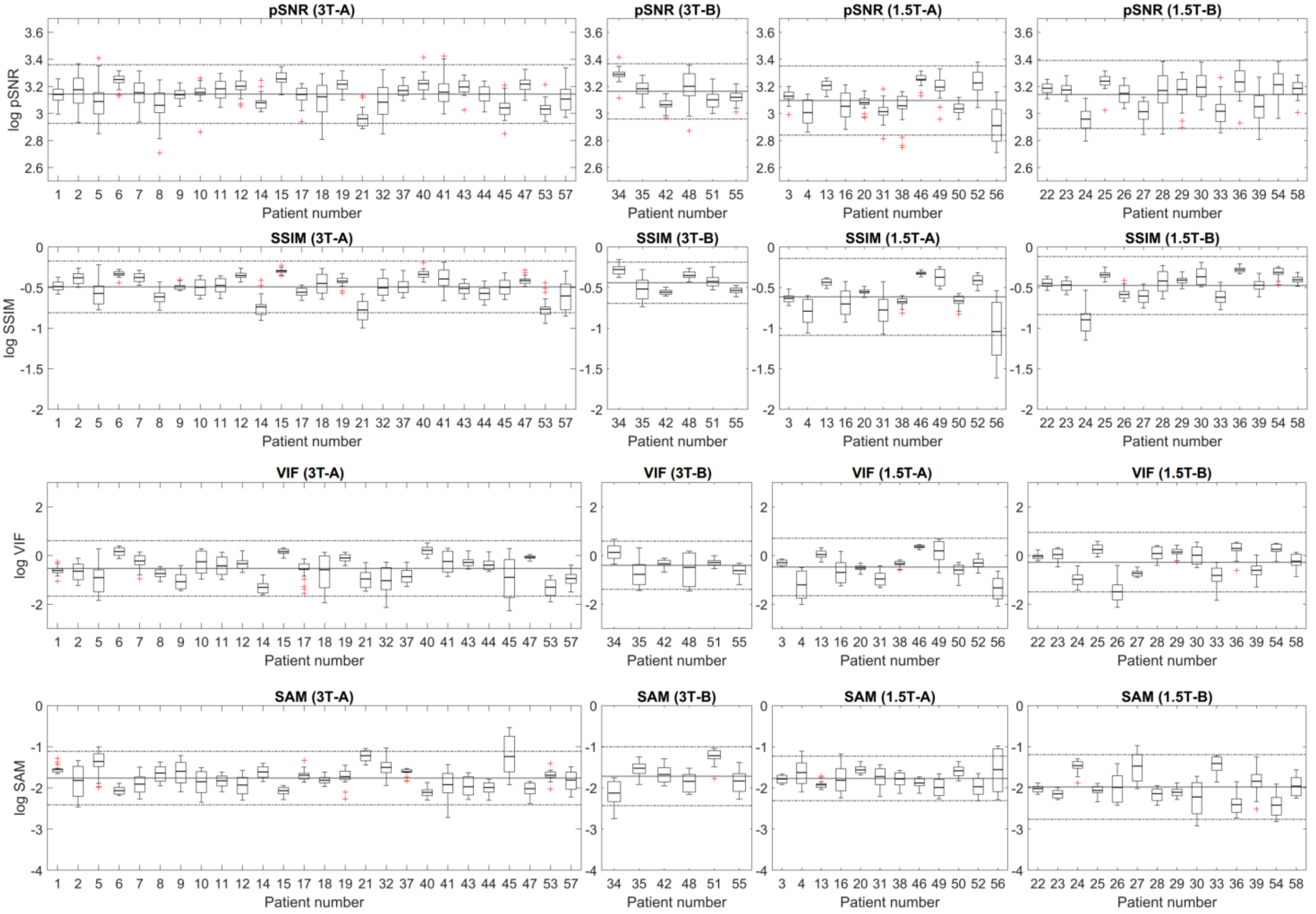

Supplementary material 3: Selected IQMs separated by MRI scanner. Paired (full-reference) image quality metrics (IQMs) for each MRI system are plotted. Box plots displaying the mean, inter quartile range, minima, maxima, and outliers were calculated for each individual patient and show the value of the IQMs comparing the axial images with and without AI-based reconstructions. Four different MR systems were included in the evaluation, labelled as 1.5T-A (n=12), 1.5T-B (n=14), 3T-A (n=26), and 3T-B (n=6), respectively. These systems were either a 1.5T MAGNETOM Sola or 3T MAGNETOM Vida (Siemens Healthineers, Erlangen, Germany). Four paired IQMs are shown here: peak signal-to-noise ratio (pSNR), structural similarity index (SSIM), visual information fidelity (VIF), and spectral angle mapper (SAM). Solid horizontal lines indicate the mean IQM value for each MR system, while dashed lines indicate ±2 standard deviations.

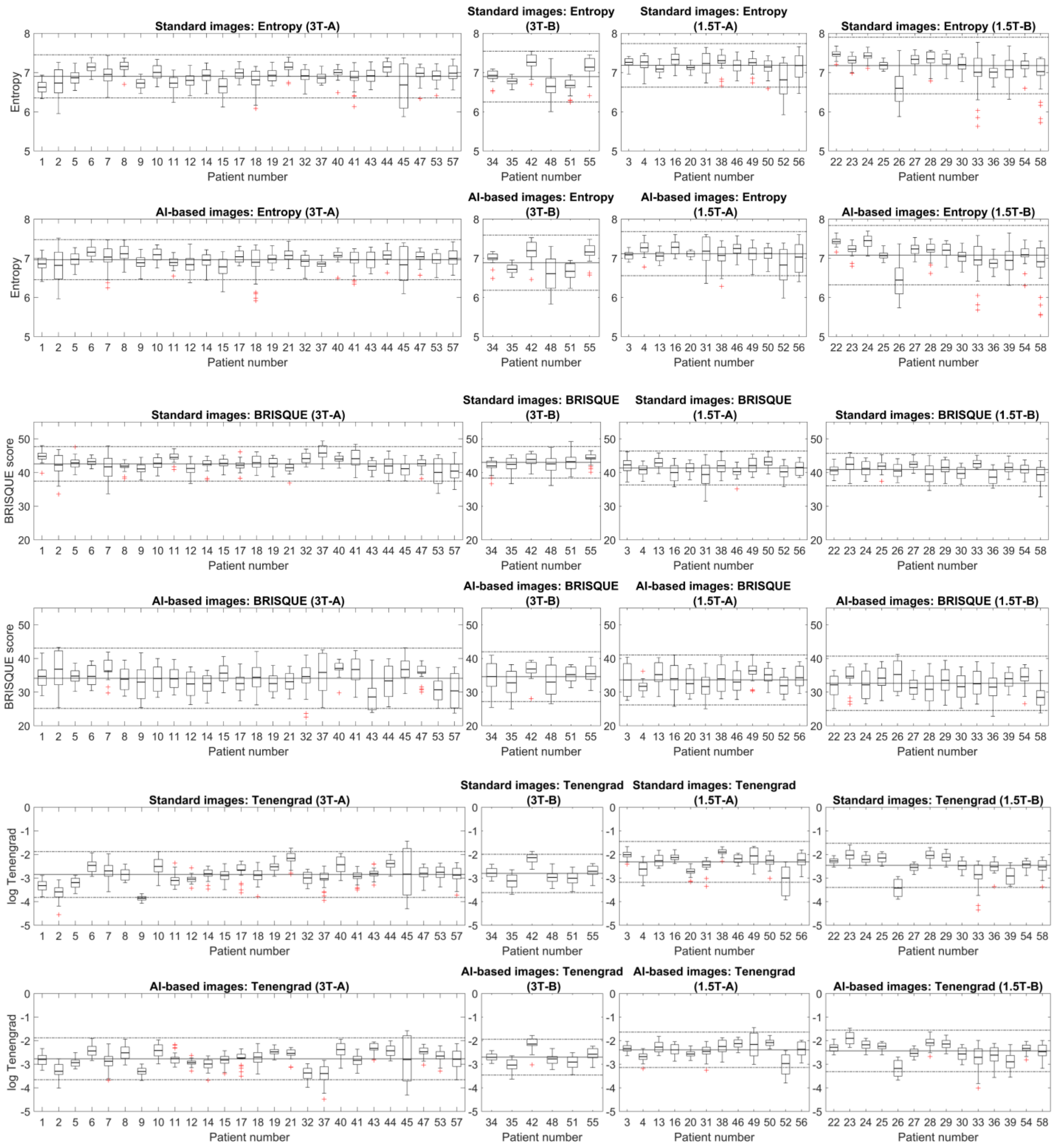

Supplementary material 4: IQMs separated by MRI scanner. Unpaired (no-reference) image quality metrics (IQMs) for each MRI system are plotted. Box plots displaying the mean, inter quartile range, minima, maxima, and outliers were calculated for each individual patient and show the value of the IQMs comparing the axial images with and without AI-based reconstructions. Four different MR systems were included in the evaluation, labelled as 1.5T-A (n=12), 1.5T-B (n=14), 3T-A (n=26), and 3T-B (n=6), respectively. These systems were either a 1.5T MAGNETOM Sola or 3T MAGNETOM Vida (Siemens Healthineers, Erlangen, Germany). Three unpaired IQMs were calculated: textural features (entropy), Blind/Referenceless Image Spatial Quality Evaluator (BRISQUE), and Tenengrad. Solid horizontal lines indicate the mean IQM value for each MR system, while dashed lines indicate ±2 standard deviations.

Supplementary material 5: Results of the pairwise comparisons between MR systems. Indicators ** are appended to the p-values < 0.05 following a Benjamini–Hochberg correction.

Image quality metric	Comparison	Estimate	Standard error	t-statistic	p-value	BH corrected p-value
Mean squared error (MSE)	3T-A vs 3T-B	0.0938	0.1981	0.4733	0.9646	1.0000
	1.5T-B vs 1.5T-A	-0.2239	0.1721	-1.3015	0.5660	1.0000
	3T-A vs 1.5T-B	-0.2239	0.1527	-1.4668	0.4644	1.0000
	3T-A vs 1.5T-A	0.0000	0.1449	0.0002	1.0000	1.0000
	1.5T-A vs 3T-B	0.3177	0.2187	1.4523	0.4730	1.0000
	1.5T-B vs 3T-B	0.0937	0.2134	0.4392	0.9714	1.0000
Peak signal-to-noise ratio (pSNR)	3T-A vs 3T-B	-0.0179	0.0383	-0.4674	0.9659	1.0000
	1.5T-B vs 1.5T-A	0.0430	0.0332	1.2947	0.5703	1.0000
	3T-A vs 1.5T-B	0.0451	0.0295	1.5277	0.4284	1.0000
	3T-A vs 1.5T-A	0.0020	0.0280	0.0721	0.9999	1.0000
	1.5T-A vs 3T-B	-0.0629	0.0423	-1.4895	0.4508	1.0000
	1.5T-B vs 3T-B	-0.0199	0.0412	-0.4828	0.9626	1.0000
Structual similarity index (SSIM)	3T-A vs 3T-B	-0.0529	0.0699	-0.7556	0.8739	1.0000
	1.5T-B vs 1.5T-A	0.1415	0.0608	2.3299	0.1039	0.6232
	3T-A vs 1.5T-B	0.1186	0.0539	2.2008	0.1360	0.6368
	3T-A vs 1.5T-A	-0.0229	0.0512	-0.4480	0.9697	1.0000
	1.5T-A vs 3T-B	-0.1715	0.0772	-2.2205	0.1307	0.6368
	1.5T-B vs 3T-B	-0.0299	0.0754	-0.3971	0.9786	1.0000
Visual information fidelity (VIF)	3T-A vs 3T-B	-0.0806	0.1450	-0.5559	0.9445	1.0000
	1.5T-B vs 1.5T-A	0.1562	0.1260	1.2399	0.6046	1.0000
	3T-A vs 1.5T-B	-0.0623	0.1117	-0.5576	0.9441	1.0000
	3T-A vs 1.5T-A	-0.2185	0.1061	-2.0589	0.1798	0.7053
	1.5T-A vs 3T-B	-0.0183	0.1601	-0.1143	0.9995	1.0000
	1.5T-B vs 3T-B	0.1379	0.1562	0.8824	0.8139	1.0000
Spectral angle mapper (SAM)	3T-A vs 3T-B	-0.0487	0.1177	-0.4133	0.9760	1.0000
	1.5T-B vs 1.5T-A	-0.2075	0.1023	-2.0290	0.1902	0.7185
	3T-A vs 1.5T-B	0.0153	0.0907	0.1686	0.9983	1.0000
	3T-A vs 1.5T-A	0.2228	0.0861	2.5862	0.0583	0.3966
	1.5T-A vs 3T-B	-0.0640	0.1300	-0.4920	0.9606	1.0000
	1.5T-B vs 3T-B	-0.2714	0.1268	-2.1401	0.1536	0.6812
Bhattacharyya distance (BD)	3T-A vs 3T-B	-0.0867	0.2200	-0.3940	0.9790	1.0000
	1.5T-B vs 1.5T-A	-0.3274	0.1911	-1.7131	0.3270	1.0000
	3T-A vs 1.5T-B	-0.0396	0.1696	-0.2337	0.9955	1.0000
	3T-A vs 1.5T-A	0.2878	0.1610	1.7876	0.2904	0.9256
	1.5T-A vs 3T-B	-0.0471	0.2430	-0.1938	0.9974	1.0000
	1.5T-B vs 3T-B	-0.3745	0.2370	-1.5798	0.3986	1.0000
Bhattacharyya coefficient (BC)	3T-A vs 3T-B	0.0005	0.0016	0.3034	0.9902	1.0000
	1.5T-B vs 1.5T-A	0.0013	0.0014	0.9358	0.7858	1.0000
	3T-A vs 1.5T-B	-0.0002	0.0013	-0.1461	0.9989	1.0000
	3T-A vs 1.5T-A	-0.0015	0.0012	-1.2649	0.5889	1.0000
	1.5T-A vs 3T-B	0.0007	0.0018	0.3768	0.9816	1.0000
	1.5T-B vs 3T-B	0.0020	0.0018	1.1407	0.6663	1.0000
Chi-square distance (χ^2D)	3T-A vs 3T-B	0.0087	0.1937	0.0448	1.0000	1.0000
	1.5T-B vs 1.5T-A	-0.2580	0.1683	-1.5334	0.4250	1.0000
	3T-A vs 1.5T-B	-0.1152	0.1493	-0.7715	0.8669	1.0000
	3T-A vs 1.5T-A	0.1428	0.1417	1.0078	0.7455	1.0000
	1.5T-A vs 3T-B	0.1239	0.2139	0.5790	0.9380	1.0000
	1.5T-B vs 3T-B	-0.1342	0.2087	-0.6429	0.9176	1.0000
Frobenius norm (FN)	3T-A vs 3T-B	0.0469	0.0991	0.4733	0.9646	1.0000
	1.5T-B vs 1.5T-A	-0.1120	0.0860	-1.3015	0.5660	1.0000
	3T-A vs 1.5T-B	-0.1120	0.0763	-1.4668	0.4644	1.0000
	3T-A vs 1.5T-A	0.0000	0.0725	0.0002	1.0000	1.0000
	1.5T-A vs 3T-B	0.1588	0.1094	1.4523	0.4730	1.0000
	1.5T-B vs 3T-B	0.0469	0.1067	0.4392	0.9714	1.0000
weighted Jaccard similarity (WJS)	3T-A vs 3T-B	0.0043	0.0177	0.2420	0.9950	1.0000
	1.5T-B vs 1.5T-A	0.0201	0.0154	1.3060	0.5632	1.0000
	3T-A vs 1.5T-B	0.0003	0.0137	0.0212	1.0000	1.0000
	3T-A vs 1.5T-A	-0.0198	0.0130	-1.5283	0.4280	1.0000
	1.5T-A vs 3T-B	0.0040	0.0196	0.2043	0.9969	1.0000
	1.5T-B vs 3T-B	0.0241	0.0191	1.2624	0.5905	1.0000
Bray-Curtis dissimilarity (BCD)	3T-A vs 3T-B	-0.0288	0.0983	-0.2925	0.9912	1.0000
	1.5T-B vs 1.5T-A	-0.1593	0.0854	-1.8654	0.2550	0.8389
	3T-A vs 1.5T-B	-0.0213	0.0758	-0.2816	0.9921	1.0000
	3T-A vs 1.5T-A	0.1380	0.0719	1.9184	0.2325	0.7906
	1.5T-A vs 3T-B	-0.0074	0.1086	-0.0684	0.9999	1.0000
	1.5T-B vs 3T-B	-0.1668	0.1059	-1.5741	0.4018	1.0000
BRISQUE (standard reconstruction)	3T-A vs 3T-B	-0.4957	0.5797	-0.8550	0.8278	1.0000
	1.5T-B vs 1.5T-A	-0.4238	0.5035	-0.8418	0.8343	1.0000
	3T-A vs 1.5T-B	1.2291	0.4468	2.7512	0.0391	0.3326
	3T-A vs 1.5T-A	1.6530	0.4237	3.9010	0.0015	**0.0379
	1.5T-A vs 3T-B	-1.7248	0.6403	-2.6937	0.0451	0.3536
	1.5T-B vs 3T-B	-2.1486	0.6244	-3.4409	0.0060	0.1024
BRISQUE (AI-based reconstruction)	3T-A vs 3T-B	-0.6079	0.8621	-0.7052	0.8946	1.0000
	1.5T-B vs 1.5T-A	-1.0008	0.7487	-1.3367	0.5440	1.0000
	3T-A vs 1.5T-B	0.4304	0.6644	0.6478	0.9159	1.0000
	3T-A vs 1.5T-A	1.4312	0.6299	2.2723	0.1174	0.6368
	1.5T-A vs 3T-B	-1.0383	0.9524	-1.0903	0.6969	1.0000
	1.5T-B vs 3T-B	-2.0391	0.9286	-2.1960	0.1374	0.6368
Entropy (standard reconstruction)	3T-A vs 3T-B	-0.0012	0.0822	-0.0151	1.0000	1.0000
	1.5T-B vs 1.5T-A	-0.0024	0.0714	-0.0335	1.0000	1.0000
	3T-A vs 1.5T-B	-0.2802	0.0633	-4.4240	0.0003	**0.0092
	3T-A vs 1.5T-A	-0.2778	0.0601	-4.6222	0.0001	**0.0070
	1.5T-A vs 3T-B	0.2790	0.0908	3.0736	0.0169	0.1729
	1.5T-B vs 3T-B	0.2766	0.0885	3.1239	0.0148	0.1675
Entropy (AI-based reconstruction)	3T-A vs 3T-B	0.0723	0.0785	0.9218	0.7933	1.0000
	1.5T-B vs 1.5T-A	-0.0386	0.0681	-0.5657	0.9418	1.0000
	3T-A vs 1.5T-B	-0.1532	0.0605	-2.5341	0.0659	0.4199
	3T-A vs 1.5T-A	-0.1147	0.0574	-1.9989	0.2011	0.7327
	1.5T-A vs 3T-B	0.2256	0.0867	2.6030	0.0561	0.3966
	1.5T-B vs 3T-B	0.1870	0.0845	2.2125	0.1328	0.6368
Tenengrad (standard reconstruction)	3T-A vs 3T-B	-0.0026	0.0118	-0.2222	0.9961	1.0000
	1.5T-B vs 1.5T-A	-0.0133	0.0102	-1.3017	0.5659	1.0000
	3T-A vs 1.5T-B	-0.0421	0.0091	-4.6378	0.0001	**0.0070
	3T-A vs 1.5T-A	-0.0288	0.0086	-3.3393	0.0081	0.1030
	1.5T-A vs 3T-B	0.0395	0.0130	3.0356	0.0188	0.1741
	1.5T-B vs 3T-B	0.0262	0.0127	2.0617	0.1788	0.7053
Tenengrad (AI-based reconstruction)	3T-A vs 3T-B	-0.0043	0.0106	-0.4014	0.9779	1.0000
	1.5T-B vs 1.5T-A	-0.0028	0.0092	-0.3085	0.9897	1.0000
	3T-A vs 1.5T-B	-0.0290	0.0082	-3.5552	0.0043	0.0876
	3T-A vs 1.5T-A	-0.0262	0.0078	-3.3786	0.0072	0.1030
	1.5T-A vs 3T-B	0.0248	0.0117	2.1177	0.1605	0.6822
	1.5T-B vs 3T-B	0.0219	0.0114	1.9218	0.2312	0.7906

Supplementary material 6: Definitions of image quality metrics included in the evaluation. Metrics are used to assess an image (I) with a total of N pixels in an m by n array. For full-reference metrics, I is compared to a reference image $(\hat{I})$ of the same dimensions. Here, $I_{i,j}$ and $\hat{I}_{i,j}$ are corresponding pixels of the test and reference image. References for each metric can be found in the main text. A test MATLAB script, which contains example metric calculations, can be found in Supplementary material 7.

Metric	Definition	Reference
Mean squared error (MSE)	$\mathrm{MSE}(I,\hat{I}) = \frac{1}{N}\sum_{i=1}^{m}\sum_{j=1}^{n}(I_{ij} - \hat{I}_{ij})^2$<br><br>Note the "`immse`$(\hat{I}, I)$" function in the MATLAB image processing toolbox [38] was used.	[30,38]
Peak signal-to-noise ratio (pSNR)	$\mathrm{pSNR}(I,\hat{I}) = 10\log_{10}\frac{\max(\hat{I})^2}{\mathrm{MSE}(I,\hat{I})}$<br><br>Note the "`psnr`$(\hat{I}, I)$" function in the MATLAB image processing toolbox [38] was used.	[30,38]
Structural similarity index (SSIM)	$\mathrm{SSIM}(I,\hat{I}) = \frac{(2\mu_I\mu_{\hat{I}} + c_1)(2\sigma_{I,\hat{I}} + c_2)}{(\mu_I^2 + \mu_{\hat{I}}^2 + c_1)(\sigma_I^2 + \sigma_{\hat{I}}^2 + c_2)}$<br><br>With:<br>• μ_I the mean of I<br>• σ_I^2 the variance of I<br>• $\sigma_{I,\hat{I}}$ the covariance of I and $\hat{I}$<br>• $c_1 = (k_1D)^2, c_2 = (k_2D)^2$<br>Where:<br>• k_1 is 0.01 and k_2 is 0.03 by default<br>• D is the dynamic range<br><br>Note the "`ssim`$(\hat{I}, I)$" function in the MATLAB image processing toolbox [38] was used.	[30,38,39]
Visual information fidelity (VIF)	Please refer to the original publication due to complexity. MATLAB code can be found in https://live.ece.utexas.edu/research/Quality/index_algorithms.htm [accessed 03/12/2024]	[40]
Spectral angle mapper (SAM)	$\mathrm{SAM}(I,\hat{I}) = \cos^{-1}\left(\frac{I \cdot \hat{I}}{\lVert I\rVert \cdot \lVert \hat{I}\rVert}\right)$<br><br>Here, each 2D image ($I$ and $\hat{I}$) have been reshaped into a 1D vector of pixel intensities.	[41]
Bhattacharyya coefficient (BC) and distance (BD)	$\mathrm{BC}(I,\hat{I}) = \sum_{i=1}^{N}\sqrt{I_i \cdot \hat{I}_i}$<br><br>$\mathrm{BD} = -\ln(\mathrm{BC})$<br><br>Here, each 2D image ($I$ and $\hat{I}$) have been reshaped and normalised into 1D vector of pixel intensities.	[42,43]
Chi-square distance (χ^2D)	$\chi^2\mathrm{D}(I,\hat{I}) = \frac{\sum_{i=1}^{N}(I_i - \hat{I}_i)^2}{\sum_{i=1}^{N}(I_i + \hat{I}_i)}$<br><br>Here, each 2D image ($I$ and $\hat{I}$) have been reshaped into a 1D vector of pixel intensities.	[44]
Frobenius norm (FN)	$\mathrm{FN}(I,\hat{I}) = \frac{1}{\sqrt{N}}\sqrt{\sum_{j=1}^{N}(I_i - \hat{I}_i)^2}$<br><br>Here, each 2D image ($I$ and $\hat{I}$) have been reshaped into a 1D vector of pixel intensities.	[45]
Weighted Jaccard similarity (WJS)	$\mathrm{WJS}(I,\hat{I}) = \frac{\sum_{i=1}^{N}\min(I_i,\hat{I}_i)}{\sum_{i=1}^{N}\max(I_i,\hat{I}_i)}$<br><br>Here, each 2D image ($I$ and $\hat{I}$) have been reshaped into a 1D vector of pixel intensities.	[46]
Bray-Curtis dissimilarity (BCD)	$\mathrm{BCD}(I,\hat{I}) = \frac{\sum_{i=1}^{N}\lvert I_i - \hat{I}_i\rvert}{\sum_{i=1}^{N}I_i + \hat{I}_i}$<br><br>Here, each 2D image ($I$ and $\hat{I}$) have been reshaped into a 1D vector of pixel intensities.	[47]
Entropy	$\mathrm{Entropy}(I) = -\sum_{k=1}^{D}p_k(I)\log_2(p_k(I))$<br><br>Here, $p_k(I) = \frac{h_k(I)}{N}$, where $h_k(I)$ is the count of pixels in image I at intensity k, N is the total number of pixels in the image, and D is the dynamic range. Note the "`entropy`(I)" function in the MATLAB image processing toolbox [38] was used.	[38]
Blind/Referenceless Image Spatial Quality Evaluator (BRISQUE)	Please refer to the original publication due to complexity. Note the "`brisque`(I)" function in the MATLAB image processing toolbox [38] was used.	[38,48]
Tenengrad	$\mathrm{Tenengrad}(I) = \frac{1}{N}\sum_{i=1}^{m}\sum_{j=1}^{n}(\nabla_x I_{ij}^2 + \nabla_y I_{ij}^2)$<br><br>Here, $\nabla_{(x,y)}$ is the gradient in the x- or y-direction. MATLAB code can be found in https://www.mathworks.com/matlabcentral/fileexchange/27314-focus-measure [accessed 03/12/2024]	[30,49]
